# Mendelian randomization analysis identified genes pleiotropically associated with central corneal thickness

**DOI:** 10.1186/s12864-021-07860-3

**Published:** 2021-07-07

**Authors:** Zhikun Yang, Jingyun Yang, Di Liu, Weihong Yu

**Affiliations:** 1 Department of Ophthalmology, State Key Laboratory of Complex Severe and Rare Diseases, Peking Union Medical College Hospital, Key Laboratory of Ocular Fundus Diseases, Chinese Academy of Medical Sciences, Beijing, China; 2 Rush Alzheimer’s Disease Center, Rush University Medical Center, Chicago, IL USA; 3 Department of Neurological Sciences, Rush University Medical Center, Chicago, IL USA; 4 Beijing Key Laboratory of Clinical Epidemiology, School of Public Health, Capital Medical University, Beijing, China

**Keywords:** Corneal thickness, Expression quantitative trait loci, Summary Mendelian randomization, Genome-wide association study

## Abstract

**Objective:**

To prioritize genes that were pleiotropically or potentially causally associated with central corneal thickness (CCT).

**Methods:**

We applied the summary data-based Mendelian randomization (SMR) method integrating summarized data of genome-wide association study (GWAS) on CCT and expression quantitative trait loci (eQTL) data to identify genes that were pleiotropically associated with CCT. We performed separate SMR analysis using CAGE eQTL data and GTEx eQTL data. SMR analyses were done for participants of European and East Asian ancestries, separately.

**Results:**

We identified multiple genes showing pleiotropic association with CCT in the participants of European ancestry. *CLIC3* (ILMN_1796423; P_SMR_ = 4.15 × 10^− 12^), *PTGDS* (ILMN_1664464; P_SMR_ = 6.88 × 10^− 9^) and *C9orf142* (ILMN_1761138; P_SMR_ = 8.09 × 10^− 9^) were the top three genes using the CAGE eQTL data, and *RP11-458F8.4* (ENSG00000273142.1; P_SMR_ = 5.89 × 10^− 9^), *LCNL1* (ENSG00000214402.6; P_SMR_ = 5.67 × 10^− 8^), and *PTGDS* (ENSG00000107317.7; P_SMR_ = 1.92 × 10^− 7^) were the top three genes using the GTEx eQTL data. No genes showed significantly pleiotropic association with CCT in the participants of East Asian ancestry after correction for multiple testing.

**Conclusion:**

We identified several genes pleiotropically associated with CCT, some of which represented novel genes influencing CCT. Our findings provided important leads to a better understanding of the genetic factors influencing CCT, and revealed potential therapeutic targets for the treatment of primary open-angle glaucoma and keratoconus.

**Supplementary Information:**

The online version contains supplementary material available at 10.1186/s12864-021-07860-3.

## Introduction

The cornea is a highly collagenous and transparent tissue through which light reaches the interior structures of the eye. Previous studies highlighted the importance of central corneal thickness (CCT) in relation to several ocular and non-ocular conditions. For example, decrease in CCT is significantly associated with intraocular pressure (IOP) [1]. Thinner CCT has been demonstrated as an important feature of keratoconus and a risk factor for primary open-angle glaucoma (POAG) in patients with ocular hypertension [2–7]. Keratoconus is the leading cause of corneal transplants worldwide, with an estimated prevalence of 0.14% [8], while POAG is the most common cause of irreversible blindness worldwide, accounting for approximately 70% of all the cases of glaucoma [9].

Epidemiologic studies have shown that CCT differs among ethnic groups, with Europeans having higher CCT values than Africans, and Asians showing a larger variation in CCT [10]. CCT is highly heritable, with an estimated heritability ranging from 68 to 95% [11–13]. Previous genome-wide association studies (GWAS) identified a number of CCT-associated loci in Europeans and Asians, such as genetic loci in *ZNF469*, *FOXO1*, *LRRK1* and *IBTK* [14–20]. Recent genetic studies revealed additional novel loci associated with CCT, some of which conferred relatively high risks of keratoconus and POAG, highlighting the potential involvement of CCT-associated genes underlying the pathogenesis of keratoconus and POAG [21, 22].

Mendelian randomization (MR) uses genetic variants as the proxy to randomization and is a promising tool to explore pleiotropic/potentially causal effect of an exposure (e.g., gene expression) on the outcome (e.g., CCT) [23]. MR could reduce confounding and reverse causation that are commonly encountered in traditional association studies, and has been successful in identifying gene expression probes or DNA methylation loci that are pleiotropically/potentially causally associated with various phenotypes, such as neuropathologies of Alzheimer’s disease and severity of COVID-19 [24, 25].

In this paper, we applied the summary data-based MR (SMR) method integrating summarized GWAS data for CCT and cis- eQTL (expression quantitative trait loci) data to prioritize genes that are pleiotropically/potentially causally associated with CCT.

## Methods

### Data sources

#### eQTL data

In the SMR analysis, cis-eQTL genetic variants were used as the instrumental variables (IVs) for gene expression. We performed SMR analysis using gene expression in blood due to unavailability of eQTL data for the eye. Specifically, we used the CAGE eQTL summarized data for blood [26], which included 2765 participants, and the V7 release of the GTEx eQTL summarized data for blood [27], which included 338 participants. The eQTL data can be downloaded at https://cnsgenomics.com/data/SMR/#eQTLsummarydata.

#### GWAS data for corneal thickness

The GWAS summarized data for CCT were provided by a recent genome-wide association meta-analysis of CCT [21]. The results were based on meta-analyses of 1000 genomes phase 1 imputed GWASs on CCT, with a total of 19 cohorts from the International Glaucoma Genetics consortium (IGGC). Specifically, the meta-analysis for participants of European ancestry included 14 cohorts with a sample of size of 17,803, and the meta-analysis for participants of East Asian ancestry included 5 cohorts with a sample size of 8107. All participating studies assumed an additive genetic model, adjusting for age, sex and at least the first five principal components. The GWAS summarized data can be downloaded at https://datashare.is.ed.ac.uk/handle/10283/2976.

#### SMR analysis

We conducted the SMR analysis with cis-eQTL as the IV, gene expression as the exposure, and CCT as the outcome. The analysis was done using the method as implemented in the software SMR. Detailed information regarding the SMR method was reported in a previous publication [28]. In brief, SMR applies the principles of MR to jointly analyze GWAS and eQTL summary statistics in order to test for pleiotropic association between gene expression and a trait due to a shared and potentially causal variant at a locus. In SMR, the top cis-eQTL was used to estimate the effect of gene expression on the outcome. SMR analysis relies on the validity of the genetic variants as IVs to obtain consistent estimates. A genetic variant has to satisfy three assumptions to be a valid IV: 1) it is associated with the risk factor (gene expression); 2) it is not associated with any confounder of the risk factor-outcome association; and 3) it is conditionally independent of the outcome given the risk factor and confounders. It was found that if the *P*-value in a linear regression of the eQTL analysis for each variant is less than 1 × 10^− 5^, then weak instrument bias was small [29]. In the SMR analysis, we adopted the default threshold of P_eQTL_ = 5 × 10^− 8^ to select the top associated cis-eQTL (with a default window size of 2000 Kb) for the SMR analysis, minimizing the risk of weak IV. By default, we removed SNPs with allele frequency difference > 0.2 between any pairwise data sets, including the LD reference sample data, the eQTL summary data and the GWAS summary data. We also conducted the heterogeneity in dependent instruments (HEIDI) test to evaluate the existence of linkage in the observed association. Rejection of the null hypothesis (i.e., P_HEIDI_ < 0.05) indicates that the observed association could be due to two distinct genetic variants in high linkage disequilibrium with each other. In conducting the HEIDI test, we adopted the default settings: removing SNPs in very strong linkage disequilibrium [LD, *r*^2^ > 0.9] with the top associated eQTL, and SNPs in low LD or not in LD [*r*^2^ < 0.05] with the top associated eQTL; P_eQTL_ < 1.5 × 10^− 3^; number of cis-SNPs≥3 for a HEIDI as HEIDI test loses power if cis-SNPs< 3; and maximum eQTLs in a HEIDI test = 20. We used false discovery rate (FDR) to adjust for multiple testing.

Data curation and statistical/bioinformatical analysis was performed using R version 4.0.3 (https://www.r-project.org/), PLINK 1.9 (https://www.cog-genomics.org/plink/1.9/) and SMR (https://cnsgenomics.com/software/smr/).

## Results

### Basic information of the summarized data

The number of participants of the CAGE eQTL data is much larger than that of the GTEx eQTL data, so is the number of eligible probes. The sample size of the GWAS data for the European ancestry is much large than that for the East Asian ancestry, so is the number of eligible genetic variants. The detailed information was shown in Table 1.

**Table 1:** Basic information of the eQTL and GWAS data

Data Source	Total # of participants	Number of eligible genetic variants or probes
**European**		
**eQTL data**		
**CAGE**	2765	16,363
**GTEx**	338	9765
**GWAS data**	17,803	815,4261
**Asian**		
**eQTL data**		
**CAGE**	2765	12,034
**GTEx**	338	6901
**GWAS data**	8107	435,9235

*CAGE* Consortium for the Architecture of Gene Expression, *eQTL* expression quantitative trait loci, *GTEx* Genotype-Tissue Expression, *GWAS* genome-wide association studies

### SMR analysis in the European population

**Fig. 1:**
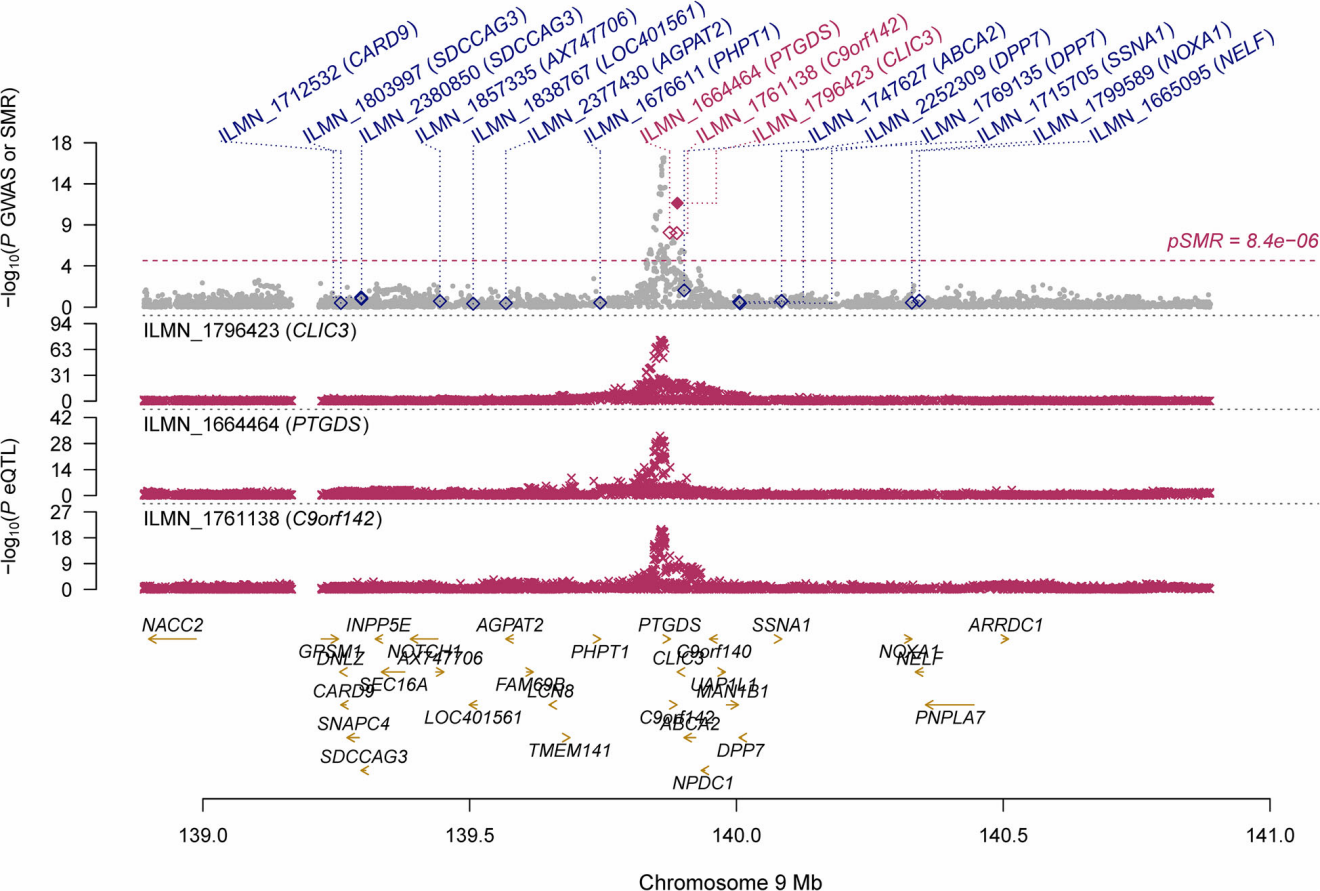
Prioritizing gene around *CLIC3*, *PTGDS* and *C9orf142* in pleiotropic association with CCT in the participants of European ancestry using CAGE eQTL data. Top plot, grey dots represent the -log_10_(*P* values) for SNPs from the GWAS of CCT, and rhombuses represent the -log_10_(*P* values) for probes from the SMR test with solid rhombuses indicating that the probes pass HEIDI test and hollow rhombuses indicating that the probes do not pass the HEIDI test. Middle plot, eQTL results. Bottom plot, location of genes tagged by the probes. *CCT* central corneal thickness, *GWAS* genome-wide association studies, *SMR* summary data-based Mendelian randomization, *HEIDI* heterogeneity in dependent instruments, *eQTL* expression quantitative trait loci

In participants with European ancestry, we identified multiple genes showing pleiotropic association with CCT after correction for multiple testing using FDR (Table 2). Specifically, using the CAGE eQTL data, our SMR analysis identified 12 genes that were pleiotropically/potentially causally associated with CCT, with *CLIC3* (ILMN_1796423; P_SMR_ = 4.15 × 10^− 12^), *PTGDS* (ILMN_1664464; P_SMR_ = 6.88 × 10^− 9^) and *C9orf142* (ILMN_1761138; P_SMR_ = 8.09 × 10^− 9^) being the top three genes (Fig. 1).

**Table 2:** Significant genes showing pleiotropic association with CCT in participants of European ancestry

eQTL data	Gene ID	Gene	CHR	Top SNP	P_**eQTL**_	P_**GWAS**_	Beta	SE	P_**SMR**_	P_**HEIDI**_	Q value
**CAGE**	ILMN_1796423	*CLIC3*	9	rs908839	8.48 × 10^−75^	6.88 × 10^−14^	6.171	0.890	4.15 × 10^−12^	2.59 × 10^− 1^	6.79 × 10^−8^
	ILMN_1664464	*PTGDS*	9	rs885070	7.83 × 10^−33^	3.43 × 10^−11^	8.474	1.463	6.88 × 10^−9^	9.97 × 10^−4^	4.41 × 10^−5^
	ILMN_1761138	*C9orf142*	9	rs4542025	1.08 × 10^−21^	4.93 × 10^− 13^	11.896	2.063	8.09 × 10^−9^	1.02 × 10^− 2^	4.41 × 10^− 5^
	ILMN_2198408	*MFF*	2	rs7560053	~ 0	1.37 × 10^−7^	−1.834	0.351	1.71 × 10^− 7^	2.36 × 10^−3^	6.99 × 10^− 4^
	ILMN_1714108	*TP53INP1*	8	rs896853	2.29 × 10^−33^	4.09 × 10^− 8^	−6.119	1.226	5.96 × 10^− 7^	1.42 × 10^−1^	1.95 × 10^−3^
	ILMN_1802894	*VKORC1L1*	7	rs2460421	4.93 × 10^−46^	3.57 × 10^− 7^	− 5.016	1.046	1.64 × 10^−6^	6.08 × 10^− 5^	4.46 × 10^−3^
	ILMN_1766859	*MSL2*	3	rs13433683	2.00 × 10^−44^	1.17 × 10^−6^	4.751	1.035	4.40 × 10^−6^	1.97 × 10^− 1^	0.010
	ILMN_1781560	*ST3GAL6*	3	rs2439225	1.80 × 10^−58^	2.04 × 10^−6^	4.043	0.887	5.23 × 10^−6^	1.83 × 10^−2^	0.011
	ILMN_1716651	*RUNX2*	6	rs1200428	8.94 × 10^−170^	5.21 × 10^−6^	2.142	0.476	6.92 × 10^−6^	1.87 × 10^−1^	0.013
	ILMN_1680171	*MFF*	2	rs7572491	3.84 × 10^−16^	1.47 × 10^−7^	−9.712	2.199	1.00 × 10^−5^	3.13 × 10^−1^	0.016
	ILMN_1785635	*BRD3*	9	rs457420	5.98 × 10^−47^	4.23 × 10^−6^	−4.571	1.043	1.18 × 10^−5^	8.29 × 10^−3^	0.018
	ILMN_2188204	*ATG12*	5	rs6872406	4.42 × 10^−61^	8.61 × 10^− 6^	3.638	0.847	1.74 × 10^−5^	8.65 × 10^−2^	0.024
**GTEx**	ENSG00000273142.1	*RP11-458F8.4*	7	rs4718424	3.54 × 10^−25^	2.02 × 10^−12^	6.017	1.034	5.89 × 10^−9^	3.58 × 10^−3^	5.76 × 10^−5^
	ENSG00000214402.6	*LCNL1*	9	rs11145951	1.84 × 10^−13^	9.02 × 10^− 16^	10.562	1.946	5.67 × 10^−8^	4.65 × 10^−1^	2.77 × 10^−4^
	ENSG00000107317.7	*PTGDS*	9	rs868102	8.79 × 10^−20^	2.18 × 10^−10^	9.142	1.756	1.92 × 10^−7^	8.55 × 10^−2^	6.24 × 10^−4^
	ENSG00000226824.2	*RP4-756H11.3*	7	rs6979488	3.99 × 10^−30^	4.92 × 10^−8^	2.943	0.598	8.66 × 10^−7^	9.24 × 10^−5^	0.002
	ENSG00000170175.6	*CHRNB1*	17	rs12600861	3.47 × 10^−17^	9.03 × 10^−7^	4.382	1.033	2.20 × 10^−5^	1.89 × 10^−1^	0.037
	ENSG00000164938.9	*TP53INP1*	8	rs2340586	1.17 × 10^−10^	1.95 × 10^−8^	− 19.873	4.694	2.30 × 10^−5^	6.53 × 10^− 1^	0.037

*The GWAS summarized data were provided by the study of Iglesias et al. and can be downloaded at https://datashare.is.ed.ac.uk/handle/10283/2976. The CAGE and GTEx eQTL data can be downloaded at https://cnsgenomics.com/data/SMR/#eQTLsummarydata

P_eQTL_ is the *P*-value of the top associated cis-eQTL in the eQTL analysis, and P_GWAS_ is the *P*-value for the top associated cis-eQTL in the GWAS analysis. Beta is the estimated effect size in SMR analysis, SE is the corresponding standard error, P_SMR_ is the *P-*value for SMR analysis and P_HEIDI_ is the *P-*value for the HEIDI test.

FDR was calculated at *P* = 10^− 3^ threshold

Bold font means statistical significance after correction for multiple testing using FDR

*CAGE* Consortium for the Architecture of Gene Expression, *CCT* central corneal thickness, *CHR* chromosome, *eQTL* expression quantitative trait loci, *GTEx* Genotype-Tissue Expression, *HEIDI* heterogeneity in dependent instruments, *SNP* single-nucleotide polymorphism, *SMR* summary data-based Mendelian randomization, *FDR* false discovery rate, *GWAS* genome-wide association studies

**Fig. 2:**
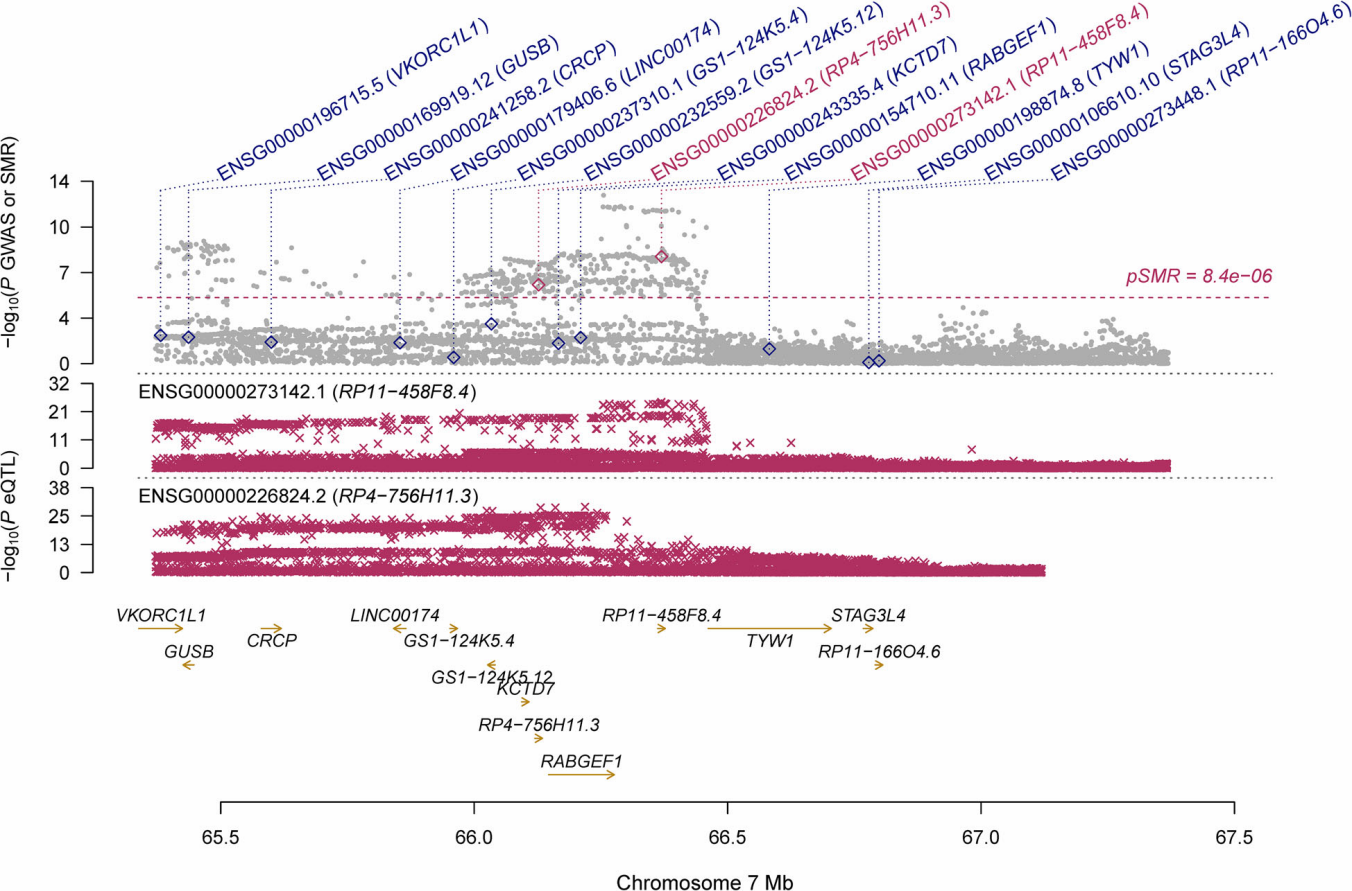
Prioritizing gene around *RP11-458F8.4* in pleiotropic association with CCT in the participants of European ancestry using GTEx eQTL data. Top plot, grey dots represent the -log_10_(*P* values) for SNPs from the GWAS of CCT, and rhombuses represent the -log_10_(*P* values) for probes from the SMR test with hollow rhombuses indicating that the probes do not pass the HEIDI test. Middle plot, eQTL results. Bottom plot, location of genes tagged by the probes. *CCT* central corneal thickness, *GWAS* genome-wide association studies, *SMR* summary data-based Mendelian randomization, *HEIDI* heterogeneity in dependent instruments, *eQTL* expression quantitative trait loci

**Fig. 3:**
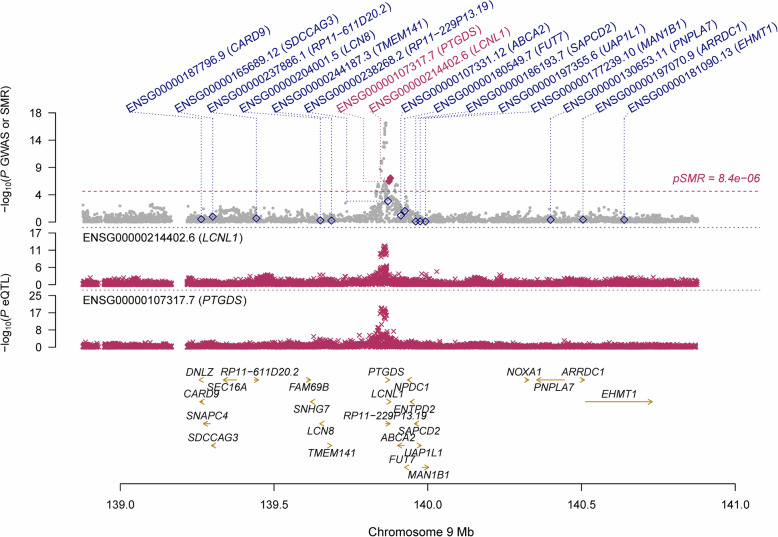
Prioritizing gene around *LCNL1* and *PTGDS* in pleiotropic association with CCT in the participants of European ancestry using CAGE eQTL data. Top plot, grey dots represent the -log_10_(*P* values) for SNPs from the GWAS of CCT, and rhombuses represent the -log_10_(*P* values) for probes from the SMR test with solid rhombuses indicating that the probes pass HEIDI test and hollow rhombuses indicating that the probes do not pass the HEIDI test. Middle plot, eQTL results. Bottom plot, location of genes tagged by the probes. *CCT* central corneal thickness, *GWAS* genome-wide association studies, *SMR* summary data-based Mendelian randomization, *HEIDI* heterogeneity in dependent instruments, *eQTL* expression quantitative trait loci

Using the GTEx eQTL data, our SMR analysis identified six genes that were pleiotropically/potentially causally associated with CCT, with *RP11-458F8.4* (ENSG00000273142.1; P_SMR_ = 5.89 × 10^− 9^; Fig. 2), *LCNL1* (ENSG00000214402.6; P_SMR_ = 5.67 × 10^− 8^; Fig. 3), and *PTGDS* (ENSG00000107317.7; P_SMR_ = 1.92 × 10^− 7^; Fig. 3) being the top three genes. It should be noted that the gene *PTGDS* was the top gene showing significant pleiotropic association in both SMR analyses.

### SMR analysis in the east Asian population

In participants of East Asian ancestry, we identified no genes showing significant pleiotropic association with CCT after correction for multiple testing using FDR (Table 3). Specifically, using the CAGE eQTL data, we found that two genes, *PASK* (ILMN_1754858, P_SMR_ = 7.98 × 10^− 5^; ILMN_1667022, P_SMR_ = 8.24 × 10^− 5^) and *HIATL1* (ILMN_1703229, P_SMR_ = 4.30 × 10^− 4^; ILMN_1737964, P_SMR_ = 5.84 × 10^− 4^), each of which was tagged by two probes, were among the top hits in the SMR analysis. However, none of the genes survived multiple comparison. Using the GTEx eQTL data, we found that several genes overlapped with the top genes in the SMR analysis using CAGE eQTL data, including *MAP 7D1*, *HIATL1*, *POLA2* and *ACADM*. Again, none of the genes survived multiple comparison.

**Table 3:** The top ten genes identified in the SMR analysis of CCT in participants of East Asian ancestry

eQTL data	Gene ID	Gene	CHR	Top SNP	P_**eQTL**_	P_**GWAS**_	Beta	SE	P_**SMR**_	P_**HEIDI**_	Q value
**CAGE**	ILMN_1754858	*PASK*	2	rs16843242	7.23 × 10^− 116^	6.20 × 10^− 5^	2.865	0.726	7.98 × 10^− 5^	3.73 × 10^− 1^	0.400
	ILMN_1667022	*PASK*	2	rs16843242	1.61 × 10^− 102^	6.20 × 10^−5^	3.056	0.776	8.24 × 10^− 5^	3.90 × 10^−1^	0.400
	ILMN_1670870	*ALCAM*	3	rs9810954	2.67 × 10^−54^	6.92 × 10^− 5^	−5.006	1.299	1.16 × 10^−4^	4.93 × 10^−1^	0.400
	ILMN_1777906	*MAP 7D1*	1	rs11263861	3.51 × 10^−109^	1.05 × 10^− 4^	−3.348	0.876	1.33 × 10^−4^	2.01 × 10^−5^	0.400
	ILMN_1778104	*ACADM*	1	rs2133134	7.36 × 10^−72^	1.85 × 10^−4^	6.341	1.733	2.52 × 10^−4^	1.15 × 10^−3^	0.575
	ILMN_1765578	*TIPARP*	3	rs344050	1.98 × 10^−9^	6.46 × 10^−6^	10.924	3.030	3.12 × 10^− 4^	3.42 × 10^−2^	0.575
	ILMN_1654065	*ATOH8*	2	rs10187314	6.31 × 10^−93^	3.22 × 10^−4^	3.393	0.958	3.97 × 10^−4^	1.95 × 10^− 1^	0.575
	ILMN_1696713	*POLA2*	11	rs487989	1.06 × 10^−30^	2.03 × 10^−4^	6.068	1.716	4.07 × 10^− 4^	1.68 × 10^− 3^	0.575
	ILMN_1703229	*HIATL1*	9	rs56987829	8.47 × 10^−26^	1.86 × 10^−4^	4.510	1.281	4.30 × 10^−4^	4.26 × 10^−2^	0.575
	ILMN_1737964	*HIATL1*	9	rs117447155	2.16 × 10^−128^	5.12 × 10^−4^	1.601	0.465	5.84 × 10^−4^	7.80 × 10^−3^	0.634
**GTEx**	ENSG00000273142.1	*RP11-458F8.4*	7	rs4718424	3.54 × 10^−25^	1.89 × 10^−6^	5.550	1.282	1.50 × 10^−4^	2.50 × 10^−2^	0.103
	ENSG00000226824.2	*RP4-756H11.3*	7	rs6979488	3.99 × 10^−30^	2.67 × 10^−5^	3.156	0.801	8.11 × 10^−4^	3.92 × 10^− 4^	0.280
	ENSG00000116871.11	*MAP 7D1*	1	rs11263861	3.60 × 10^−36^	1.05 × 10^−4^	−7.011	1.892	2.11 × 10^−4^	4.71 × 10^−3^	0.484
	ENSG00000148110.11	*HIATL1*	9	rs56074891	3.46 × 10^−33^	1.95 × 10^−4^	4.615	1.297	3.73 × 10^−4^	3.17 × 10^−2^	0.644
	ENSG00000241258.2	*CRCP*	7	rs1643388	1.34 × 10^−34^	2.99 × 10^−4^	−4.654	1.342	5.24 × 10^−4^	1.81 × 10^−10^	0.723
	ENSG00000238268.2	*RP11-229P13.19*	9	rs3814499	4.82 × 10^−36^	4.58 × 10^−4^	4.691	1.390	7.38 × 10^−4^	1.65 × 10^−1^	0.849
	ENSG00000014138.4	*POLA2*	11	rs1858783	3.68 × 10^−14^	2.52 × 10^−4^	7.358	2.233	9.82 × 10^−4^	6.70 × 10^−4^	0.941
	ENSG00000203363.2	*AC012454.4*	2	rs1561285	1.15 × 10^−19^	5.29 × 10^−4^	3.674	1.135	1.21 × 10^−3^	8.10 × 10^−1^	0.941
	ENSG00000117054.9	*ACADM*	1	rs61799967	1.32 × 10^−12^	3.69 × 10^−4^	14.740	4.631	1.46 × 10^−3^	4.90 × 10^− 3^	0.941
	ENSG00000168528.7	*SERINC2*	1	rs7541464	4.49 × 10^−25^	8.34 × 10^−4^	3.282	1.032	1.48 × 10^−3^	7.00 × 10^− 2^	0.941

*The GWAS summarized data were provided by the study of Iglesias et al. and can be downloaded at https://datashare.is.ed.ac.uk/handle/10283/2976. The CAGE and GTEx eQTL data can be downloaded at https://cnsgenomics.com/data/SMR/#eQTLsummarydata

P_eQTL_ is the *P*-value of the top associated cis-eQTL in the eQTL analysis, and P_GWAS_ is the *P-*value for the top associated cis-eQTL in the GWAS analysis. Beta is the estimated effect size in SMR analysis, SE is the corresponding standard error, P_SMR_ is the *P-*value for SMR analysis and P_HEIDI_ is the *P-*value for the HEIDI test

FDR was calculated at *P* = 10^− 3^ threshold

*CAGE* Consortium for the Architecture of Gene Expression, *CCT* central corneal thickness, *CHR* chromosome, *eQTL* expression quantitative trait loci, *GTEx* Genotype-Tissue Expression, *HEIDI* heterogeneity in dependent instruments, *SNP* single-nucleotide polymorphism, *SMR* summary data-based Mendelian randomization, *FDR* false discovery rate, *GWAS* genome-wide association studies

## Discussion

In the present study, we integrated summarized data of GWAS on CCT and eQTL data in the MR analysis to explore putative genes that showed pleiotropic/potentially causal association with CCT. In the participants of European ancestry, we identified multiple genes showing significantly pleiotropic association with CCT, some of which represented novel genes associated with CCT. Our findings provided important leads to a better understanding of the genetic factors influencing CCT, and revealed potential therapeutic targets for the treatment of POAG and keratoconus.

We found that *PTGDS* (prostaglandin D2 synthase) showed significantly pleiotropic association with CCT in the participants of European ancestry using both CAGE and GTEx eQTL data. *PTGDS* encodes the glutathione-independent prostaglandin D2 synthase which catalyzes the conversion of prostaglandin H2 (PGH2) to prostaglandin D2 (PGD2), an important marker for keratocytes [30, 31]. A genetic polymorphism in *PTGDS* (rs11145951) was reported to be associated with CCT in the European population (*P* = 9.20 × 10^− 12^) but not in the Asian population (*P* = 2.30 × 10^− 2^) [15]. The association of this polymorphism with CCT did not reach genome-wide significance in the Latino population (*P* = 1.15 × 10^− 5^), suggesting ethnic-specific effect of this genetic polymorphism on CCT [32]. *PTGDS* was found to be highly expressed in corneal endothelial cells (CECs) [33], ranked the among the top 50 most highly expressed genes in CECs [34]. When using a novel dual media culture approach for the in vitro expansion of primary human corneal endothelial cells (hCECs), the expression of *PTGDS* increased by 12.64 folds following exposure of cultivated hCECs from proliferative (M4) to maintenance (M5) medium [35]. Given that thinner CCT was an important feature for keratoconus and a potential risk factor for POAG, these findings, together with ours, demonstrated the important role of *PTGDS* in influencing CCT and highlighted the potential of this gene as a promising target for the prevention and treatment of keratoconus and POAG.

*RP11-458F8.4* showed the most significantly pleiotropic association with CCT using GTEx eQTL data in the participants of European ancestry (Table 2). *RP11-458F8.4* is a long intergenic noncoding RNA (lincRNA). LincRNAs exercise various tissue-specific functions such as remodeling chromatin and genome architecture, RNA stabilization and transcription regulation [36]. *RP11-458F8.4* was reported to be associated with various types of malignant tumors. For example, it was upregulated in late-stage colon cancers, and its expression may be involved in the progression of colon cancer [37]. It was found to be a prognostic lincRNA in high-grade serous epithelial ovarian cancer [38], and was differently expressed in patients with breast cancer [39]. No studies have reported the association of this gene with CCT. As a result, further investigation is needed to elucidate the exact functions of this gene and examine its role in influencing CCT.

*VKORC1L1* (vitamin K epoxide reductase complex subunit 1 like 1) was pleiotropically associated with CCT in the participants of European ancestry using CAGE eQTL data. *VKORC1L1* can mediate vitamin K-dependent intracellular antioxidant functions [40]. A genetic variant in *VKORC1L1* (rs11763147) was found to be associated with CCT in a combined meta-analysis of the European and the Asian samples (4.0 × 10^− 9^, 15]. Another genetic variant (rs10563220) located upstream of *VKORC1L1* was found to be significantly associated with intraocular pressure in a GWAS of 8552 Chinese participants [41]. More studies are needed to elucidate the exact functions of *VKORC1L1* in association with CCT, and explore whether/how it is involved in the pathogenesis of POAG and keratoconus.

*RUNX2* (runt-related transcription factor 2) showed significantly pleiotropic association with CCT in the participants of European ancestry using the CAGE eQTL data (Table 2). *RUNX2* is a member of the RUNX family of transcription factors and encodes a nuclear protein, a key transcription factor of osteoblast differentiation [42, 43]. A genetic variant in *RUNX2* (rs13191376) was found to be associated with CCT in a cross-ancestry GWAS [21]. Another genetic variant in *RUNX2* (rs1755056) was found to be significantly associated with IOP and weakly associated with POAG in a GWAS combing participants from the UK Biobank and published data from International Glaucoma Genetic Consortium [44]. In a transient transfection experiment using MG-63 cells and primary bovine corneal keratocytes, *RUNX2* transcription factors affected the expression of several small leucine rich proteoglycans (SLRP) including mimecan, biglycan and keratocan [45], which were shown to be important for the development and maintenance of corneal transparency [46–48]. In another study using the rabbit corneal epithelial cell line RCE1(5 T5), RNAseq based transcriptome analysis showed that *RUNX2* exhibited the highest expression in terminally differentiated corneal epithelial cells [49]. In breast cancer, it was found that *RUNX2* functioned through the androgen receptor to regulate prolactin-induced protein (PIP) [50], a new biomarker for keratoconus [51]. These findings suggested that *RUNX2* likely plays an important role in affecting CCT and the susceptibility of POAG and keratoconus.

Of the multiple genes showing significantly pleiotropic association with CCT in the participants of European ancestry, none was significant in the SMR analysis for the participants of the East Asian ancestry. And only *RP11-458F8.4*, which was pleiotropically associated with CCT in the participants of European ancestry using GTEx data, appeared among the top-hit genes in the SMR analysis for the participants of East Asian ancestry using GTEx data (Tables 2 and 3). These findings implied possible ethnicity-specific genetic mechanisms underlying CCT, leading to anatomic changes (e.g., cornea and optic nerves) [52–54] and differences in the susceptibility of CCT-related disorders/diseases. For example, the prevalence of POAG was reported to be high in Afro-Caribbeans and African Americans, intermediate in Latinos and Asians, and low in non-Hispanic whites [55]. Moreover, compared with Asians, Europeans have a higher rate of keratoconus incidence and an older age-of-onset of keratoconus [56, 57]. The findings of our study revealed genes harboring potential biomarkers for tailored screening and treatment of POAG and keratoconus in subjects of different ethnicities.

Our study has some limitations. The number of probes used in our SMR analysis was limited, especially in the SMR analysis of the participants of East Asian ancestry. Moreover, the sample size of eQTL analysis is limited, especially for the GTEx data, which may lead to reduced power in the eQTL analysis. As a result, we may have missed some important genes. Moreover, the sample size and the number of eligible genetic variants for GWAS in the participants of East Asian ancestry is limited, compared to GWAS in the participants of European ancestry, which may affect the power of our SMR analysis and contribute to the insignificant findings in the participants of East Asian ancestry. The HEIDI test was significant for some of the identified genes (Table 2-3). As results, the possibility of horizontal pleiotropy, i.e., the identified association might be due to two distinct genetic variants in high linkage disequilibrium with each other, could not be ruled out. We only used eQTL data in the blood due to the unavailability of eQTL data from the eye. Our findings need to be validated in the future when eQTL data from the eye are available. We used the same eQTL data in the SMR analysis for both European and Asian ancestry. It is possible that eQTL pattern could vary with ethnicity. Ethnicity-specific eQTL data with larger sample size are needed in future SMR research.

## Conclusions

We identified several genes pleiotropically associated with CCT, some of which represented novel genes influencing CCT. Our findings provided important leads to a better understanding of the genetic factors influencing CCT, and revealed potential therapeutic targets for the treatment of POAG and keratoconus.

## Supplementary Information

**Additional file 1.**

## Data Availability

All data generated or analyzed during this study are publicly available as specified in the methods section of this paper. Specifically, the eQTL data can be downloaded at https://cnsgenomics.com/data/SMR/#eQTLsummarydata, and the GWAS summarized data can be downloaded at https://datashare.is.ed.ac.uk/handle/10283/2976.
