# Supplementary material for: Mendelian randomization analysis identified genes pleiotropically associated with central corneal thickness
Source: BMC Genomics. 2021 Jul 7;22:517. doi: 10.1186/s12864-021-07860-3 (PMC8263012; doi:10.1186/s12864-021-07860-3)
Supplement: Supplementary file 1 — Additional file 1. [file 12864_2021_7860_MOESM1_ESM.docx]

###shell code for SMR analysis using CAGE data for European ancestry#######

#!/bin/bash

PATH=$PATH:/home/myang/temp/SMR

ethnicity="eur"

eqtl="CAGE.sparse"

#Whole_blood is the name used for GTEx and CAGE.sparse is the name used for CAGE

echo $PATH

for race in $ethnicity

do

for data in $eqtl

do

smr_Linux --bfile /san2/myang/Di_liu/SMR/data/GWAS/1000g --gwas-summary /home/myang/project/Weihong/SMR/Corneal_thickness/mygwas_ct_${race}.ma --beqtl-summary /san2/myang/project/Covid/SMR/severe/cage_eqtl_data/${data} --out /home/myang/project/Weihong/SMR/Corneal_thickness/result/smr_ct_${race}_${data} --thread-num 10

done

done

###shell code for SMR analysis using CAGE data for East Asians#######

#!/bin/bash

PATH=$PATH:/home/myang/temp/SMR

ethnicity="asian"

eqtl="CAGE.sparse"

#Whole_blood is the name used for GTEx and CAGE.sparse is the name used for CAGE

echo $PATH

for race in $ethnicity

do

for data in $eqtl

do

smr_Linux --bfile /san2/myang/Di_liu/SMR/data/GWAS/1000g --gwas-summary /home/myang/project/Weihong/SMR/Corneal_thickness/mygwas_ct_${race}.ma --beqtl-summary /san2/myang/project/Covid/SMR/severe/cage_eqtl_data/${data} --out /home/myang/project/Weihong/SMR/Corneal_thickness/result/smr_ct_${race}_${data} --thread-num 10

done

done

###shell code for SMR analysis using GTEx data for European ancestry#######

#!/bin/bash

PATH=$PATH:/home/myang/temp/SMR

ethnicity="eur"

eqtl="Whole_Blood"

#Whole_blood is the name used for GTEx and CAGE.sparse is the name used for CAGE

echo $PATH

for race in $ethnicity

do

for data in $eqtl

do

smr_Linux --bfile /san2/myang/Di_liu/SMR/data/GWAS/1000g --gwas-summary /home/myang/project/Weihong/SMR/Corneal_thickness/mygwas_ct_${race}.ma --beqtl-summary /home/myang/project/Feng_wang/CP/${eqtl} --out /home/myang/project/Weihong/SMR/Corneal_thickness/result/smr_cp_${race}_${data} --thread-num 10

done

done

###shell code for SMR analysis using GTEx data for East Asians#######

#!/bin/bash

PATH=$PATH:/home/myang/temp/SMR

ethnicity="asian"

eqtl="Whole_Blood"

#Whole_blood is the name used for GTEx and CAGE.sparse is the name used for CAGE

echo $PATH

for race in $ethnicity

do

for data in $eqtl

do

smr_Linux --bfile /home/myang/GWAS/1K_reference/EAS --gwas-summary /home/myang/project/Weihong/SMR/Corneal_thickness/mygwas_ct_${race}.ma --beqtl-summary /home/myang/project/Feng_wang/CP/${eqtl} --out /home/myang/project/Weihong/SMR/Corneal_thickness/result/smr_cp_${race}_${data}_new --thread-num 10

done

done

############R code for generating the SMR plots##################

###########This file contains the R code for generating the SMR plots

dir<-'G:/research/Weihong/SMR/Corneal_Tickness/plot/' # directory where the SMR analysis results are saved

library(tidyverse)

source(paste0(dir,"plot_SMR.r")) # the function for generating SMR plot

#######################generating SMR plot for CAGE

eqtl<-'CAGE.sparse'

fn<-c('ILMN_1796423', 'ILMN_1664464', 'ILMN_1761138', 'ILMN_2198408', 'ILMN_1714108', 'ILMN_1802894', 'ILMN_1766859', 'ILMN_1781560', 'ILMN_1716651', 'ILMN_1680171')

nm.pdf<-paste0(dir,'smr_ct_eur_',eqtl,'_',Sys.Date(),'.pdf')

pdf(nm.pdf,width=12,height=8)

for (i in 1:length(fn)){

nm.plot<-paste0(dir,'smr_ct_eur_',eqtl,'_plot.',fn[i],'.txt')

SMRData = ReadSMRData(nm.plot)

SMRLocusPlot(data=SMRData, smr_thresh=8.4e-6, heidi_thresh=0.05, plotWindow=1000, max_anno_probe=16)

}

dev.off()

#######################generating SMR plot for GTEx

eqtl<-'Whole_Blood'

fn<-c('ENSG00000273142.1', 'ENSG00000214402.6', 'ENSG00000107317.7', 'ENSG00000226824.2', 'ENSG00000170175.6', 'ENSG00000164938.9')

nm.pdf<-paste0(dir,'smr_ct_eur_',eqtl,'_',Sys.Date(),'.pdf')

pdf(nm.pdf,width=12,height=8)

for (i in 1:length(fn)){

nm.plot<-paste0(dir,'smr_ct_eur_',eqtl,'_plot.',fn[i],'.txt')

SMRData = ReadSMRData(nm.plot)

SMRLocusPlot(data=SMRData, smr_thresh=8.4e-6, heidi_thresh=0.05, plotWindow=1000, max_anno_probe=16)

}

dev.off()
